# Application of social identity models of collective action to facilitate participation in groundwater aquifer storage and recovery management

**DOI:** 10.3389/fpsyg.2022.996877

**Published:** 2022-11-09

**Authors:** Naser Valizadeh, Mehdi Bagheri-Gavkosh, Masoud Bijani, Dariush Hayati

**Affiliations:** ^1^Department of Agricultural Extension and Education, School of Agriculture, Shiraz University, Shiraz, Iran; ^2^Irrigation and Reclamation Engineering Department, College of Agriculture and Natural Resources, University of Tehran, Tehran, Iran; ^3^Department of Agricultural Extension and Education, College of Agriculture, Tarbiat Modares University (TMU), Tehran, Iran

**Keywords:** aquifer storage and recovery, water management, farmers’ participation, social identity, Fashafuyeh plain, environmental psychology

## Abstract

Aquifer storage and recovery (ASR) is considered as an innovative method and an alternative one for sustainable management of water resources that has, in recent years, attracted the attention of experts and thinkers. Implementation of this method would entail the participation and collective action of various stakeholders. In this process, farmers are considered as the most important stakeholders; and limited studies have been conducted on their intentions to participate in collective actions of ASR management. In this regard, the investigation of farmers’ intention to participate in ASR and its determinants, using social identity models of collective action, was selected as the main purpose of the present study. For this purpose, using a cross-sectional survey, 330 Iranian farmers were interviewed. In this study, the ability of the dual-pathway model of collective action (DPMCA) and the encapsulation model of social identity in collective action (EMSICA) was evaluated and compared to explain farmers’ intentions towards participation in ASR management. The results revealed that the both models had good predictive powers. However, DPMCA was a stronger framework than EMSICA for facilitating farmers’ collective behaviors in the field of participation in ASR management. This is one of the most important results of the present research that might be used by various users including decision makers, managers, and practitioners of water resources management in Iran and generally the world. Finally, the creation of a “*we thinking system*” or social identity in the field of ASR management was highlighted as one of the most important take-home messages.

## Highlights

- Aquifer storage and recovery is an innovative method for water management.
- Collective action models are useful for implementation of aquifer storage and recovery.
- Formation of social identity is a crucial for collective aquifer storage and recovery.
- The study introduces *we thinking system* as the best strategy for aquifer storage and recovery.

## Introduction

Iran has always been regarded as a dry country where only one-fourth of its surface has been suitable for agricultural practices; the remaining three quarters are covered by deserts and mountains (Madani, 2014). In Iran, the average annual rainfall has been about 250 mm in the recent three decades (Fallahati et al., 2020), which does not have a proper and fair distribution in terms of time and space (Khozeymehnezhad and Tahroudi, 2019). It is apparent that in water sector and resources management, Iran is facing many problems. Brisk population growth, migration and de-peasantization, water distribution issues, low quality of water, inefficient agriculture sector, dream of food self-reliance, increased demand for water, low price of energy and water, dams, deep wells, droughts, floods, climate change, thirst for development, sanctions and economic instability, inadequate water governance structure, and lack of environmental awareness are of the most significant reasons of this issue (Madani et al., 2016; Valizadeh et al., 2020).

Increasing population and increasing water demand; as well as shortage of water resources, have a great impact on various agricultural, industrial, and service sectors (Tizro et al., 2011); thus, overexploitation of groundwater resources has caused extreme groundwater level decline, increased pollution, and increased public concern in most plains (Arabameri et al., 2019; Ghobadi et al., 2020). Every year, owing to the migration of people to urban areas, a huge volume of wastewater is continuously produced by metropolitan areas; This water source is usually used around urban areas directly for agricultural purposes (Deh-Haghi et al., 2020) and that is prevented from being overrun into the aquifers (Khanpae et al., 2020); though, the direct consumption of this wastewater has many environmental, social, and economic consequences. In other words, by directing them into aquifers in ASR management process,such impacts might be avoided (Maliva et al., 2020; Zekri et al., 2021). ASR means directing water into the aquifer at times when water is available and also re-harvesting that when it is needed (Forghani and Peralta, 2018). With regard to the characteristics of the aquifers, this method might be used as a suitable way for water storage in arid and semi-arid areas. It can be particularly effective in areas with low water resources, severe groundwater levels, and high salinity of aquifers.

ASR, as an approach to water storage (Smith et al., 2017), is an inexpensive solution to increase water storage, to remove pollution (Stuyfzand and Osma, 2019), to reduce aquifer salinity (Ghaffour et al., 2013; Gibson et al., 2018; Sathish and Mohamed, 2018), and to improve aquifer quality (Smith et al., 2017; Ghose et al., 2018). This method has advantages such as evaporation decrease, no need for large land areas for implementation, low cost of implementation (Bouwer, 2002; Khan et al., 2008; National Research Council, 2008; Maliva and Missimer, 2010; Forghani and Peralta, 2018; Wasif and Hasan, 2020), potentiality for being used in different aquifers and climates (Maliva et al., 2011; Jeong et al., 2018), and prevention of the progression of the salinity (Pyne, 2005; Zuurbier and Stuyfzand, 2017).

Throughout the year, Municipal sewage production is referred to an accessible water source for the agricultural and industrial sectors on the outskirts of metropolitan areas. This alternative water source might be used for artificial aquifer feeding and prevention of environmental pollution; that is, to achieve sustainable management of water resources and to reduce the economic, social, and environmental problems of metropolitan areas, waste water is one of the best subjects (Sathish and Mohamed, 2018). The use of waste water for feeding aquifers removes or reduces metals (Page et al., 2017; Vanderzalm et al., 2020), improves the microbial, physical, and chemical quality of waste water (NASEM, 2016; Sharma and Kennedy, 2017), reduces the risk of diseases and health human problems, values less than wastewater treatment, and evaporates less than surface water (Nema et al., 2001; Crites et al., 2014; Sharma and Kennedy, 2017).

ASR management has the potentiality to be used in various regions such as Iran that are struggling with many problems as to excessive abstractions of groundwater and decline of groundwater levels (Bagheri et al., 2020). Fashafuyeh plain in Iran due to its unique economic, social, and geographical characteristics, has been recently considered as one of the target areas for ASR management. With drying of *Qanats* and significant decrease in the surface water of Karaj River which is caused by the construction of a dam upstream of it, the water inflow of this river to Fashafoyeh area has sharply decreased in the last few decades. This has led to the seasonality of rivers/surface currents entering the plain; besides, the agricultural sector of this region, owing to the interruption of Karaj River, relies on dam overflows, and therefore groundwater resources are not responsible for compensation of this shortage. In addition to the agricultural sector, residential sectors, airports, and large industrial hubs are applicants for having water in Fashafuyeh plain. However, the increase in water abstraction over the past few decades has severely affected the aquifer in terms of quantity and quality. Thus, most areas have EC values greater than 2000 thousand μs/cm. This has made villages down the plains face salinity problem in drinking water supply; besides, drilling wells for agricultural and industrial purposes has caused the aquifer to experience an extreme groundwater level decline.

Due to the existence of these issues in Fashafuyeh plain, it seems that ASR management might be one of the best ways for achieving sustainability in water resource management in this area; where the agricultural sector is the main consumer of water in that. Therefore, farmers are certainly considered as one of the most significant stakeholders in ASR, the behavioral intention of whom towards participation would be of great importance (Bagheri et al., 2020). In other words, farmers’ participation is one of the main prerequisites of any sustainable water resource management program (Rahimi-Feyzabad et al., 2020; Valizadeh et al., 2021a,b,c); ASR management in Fashafoyeh plain is no exception to this rule and in its implementation, the need for farmers’ participation is undeniable; However, it should be mentioned that the crisis of water resources management in Fashafuyeh plain is a regional level (not individual level) crisis and requires collective actions of stakeholders, i.e., farmers (Bagheri et al., 2020). In other words, the intention towards participation in collective actions in large-scale crises such as sustainable management of water resources in Fashafuyeh plain is much more effective than individual actions of farmers. Preliminary studies conducted around the world and Iran show that no study has been conducted to determine the intention of farmers towards participation in collective ASR management. Conducting such research might provide innovative and useful solutions, insights, and policies for managers, planners, decision-makers, and other stakeholders of sustainable water resources management. In this regard, investigating farmers’ intentions towards participation in ASR, using social identity models of collective action, was determined as the main purpose of the present study.

## Theoretical framework

Many sustainability-related issues, such as sustainable management of water resources require collective action of farmers and must be done in coordination with all farmers. For example, ASR management, integrated pest management, watershed management, and wetland management require long-term and large-scale measures. In other words, they need collective action (Meinzen-Dick and Di Gregorio, 2004). Collective action is an action taken by a group of individuals to pursue perceived common interests (Scott and Marshall, 2009; Judge et al., 2022; Sia and Wilson, 2022; Valizadeh et al., 2022; Wibisono et al., 2022). Among community members, collective action and networking is of many advantages. For instance, it facilitates access to information and allows farmers as members of the community, to participate in the development of related technologies (Valizadeh et al., 2021a,c; Odağ et al., 2022). It also makes it easier for them to access financial credit, and also share potential risks among members of the community (Meinzen-Dick and Di Gregorio, 2004; Khumalo et al., 2022). The social psychological mechanisms underlying collective actions are configured in the form of some collective identity models. The dual-pathway model of collective action (DPMCA) and the encapsulation model of social identity in collective action (EMSICA) are among these frameworks that consider the determinants influencing participation in collective action by basing social identity as a crucial determinant in this field (Shi et al., 2015).

### The dual-pathway model of collective action

**Figure 1:**
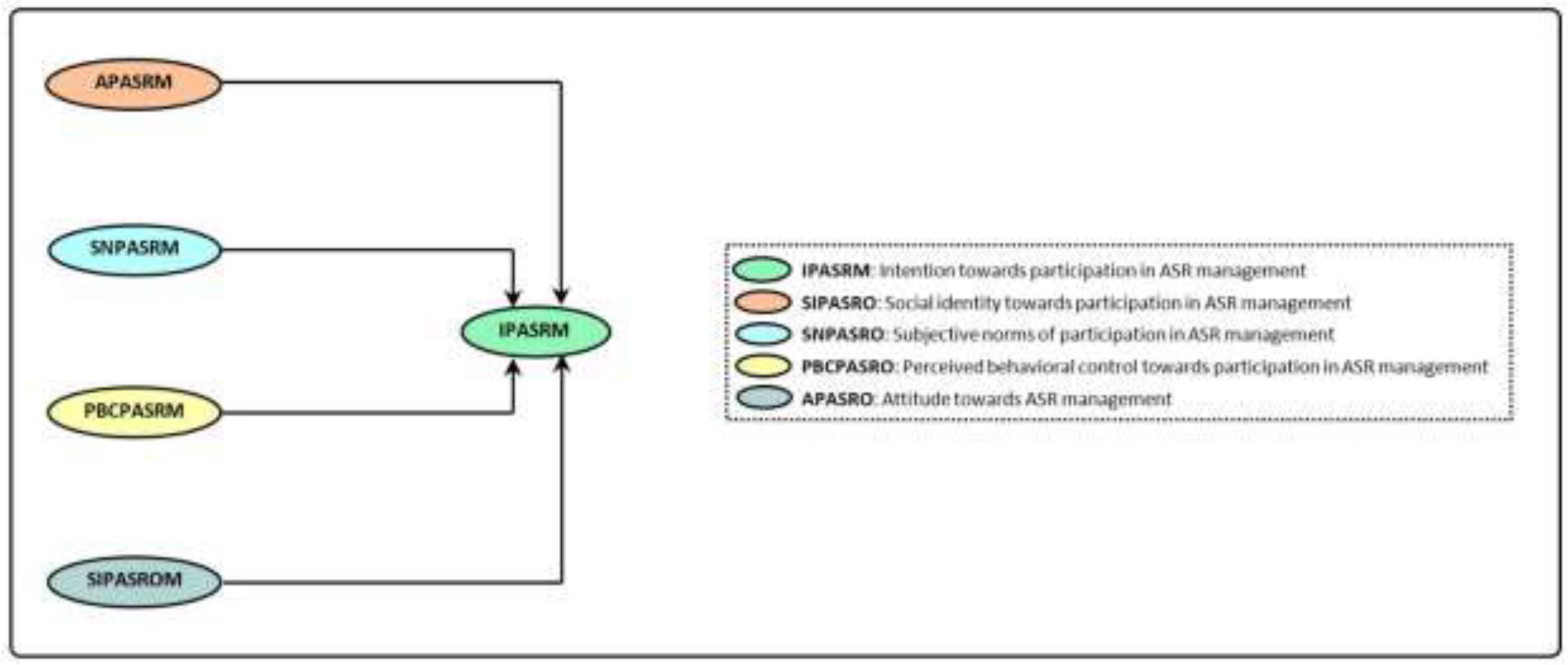
The dual-pathway model of collective action (Bamberg et al., 2015).

DPMCA was first developed by Stürmer and Simon (Stürmer and Simon, 2004, 2009; Figure 1). This model is based on two distinguished interpretations or pathways for motivating participation and collective action. The first interpretation is based on the cost–benefit assessments of individuals. In other words, people who look through the lens of cost–benefit worldview to participation in collective actions such as ASR expect external rewards. Of course, it should be mentioned that in this interpretation, the meaning of external reward refers to the outcome expectancy for collective action. In the second interpretation, social identity is considered as a concept and motivation of social movement participation behavior (Tajfel and Turner, 1986). Through participation in ASR collective actions, Identification acts as a driver in this pathway. The cost–benefit pathway was proposed by Olson (1965) and is also known as Olson’s social dilemma. According to this paradox, individuals pursue their individual and personal goals in collective and participative actions (Bamberg et al., 2015). This conflict of individual interests of group members with collective interests could in some cases lead to the failure to form genuine collective action. In the second pathway or theory of social identity, collective and group goals are considered as the ultimate goal of individual participation. For instance, if farmers participate in ASR management that is due to this fact that they feel it is beneficial to the farmer group/community and their participation strengthens the group’s position (Liao et al., 2020). It is worth mentioning that striving for collective and group goals can lead to the formation of individuals’ identities and strengthens their self-confidence in individual actions’ effectiveness, which is also crucial in determining the intentions and behavior (Scheepers and Ellemers, 2019). Furthermore, the second interpretation (social identity theory) introduces normative motives as another root of individuals’ collective behavior. This motive refers to the effect of the type of thinking of group members or social environment on individuals’ intentions and behaviors (Klandermans, 1984; Bingley et al., 2022; Valizadeh et al., 2022a). According to Bamberg et al. (2015), these three motives are combined with the Reasoned Action Theory of Ajzen and Fishbein et al. (1980) and pave the way for construction of a more comprehensive theory entitled DPMCA, in which collective identity, attitude, subjective norms of behavior, and perceived control on the behavior are the main predictors of intentions towards participation in collective pro-environmental actions (Figure 1). These four variables are in fact manifestations of three normative, reward, and collective motives.

### The encapsulation model of social identity in collective action

**Figure 2:**
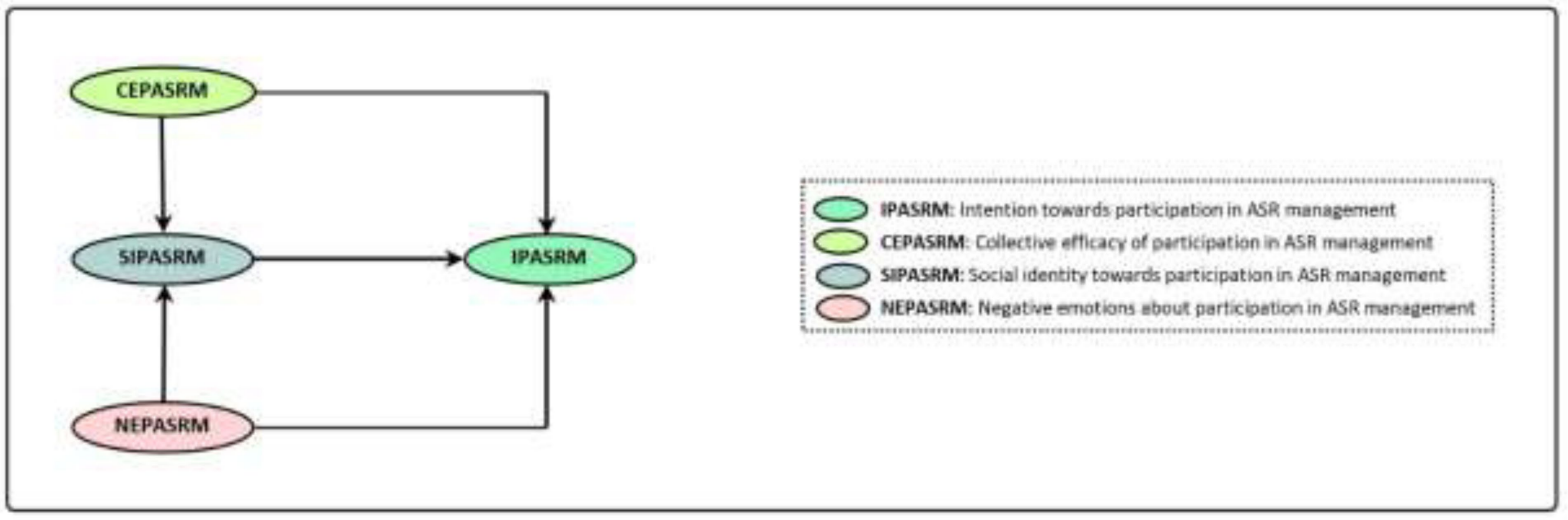
The relationships of variables in EMSICA (Thomas et al., 2009, 2012).

EMSICA was developed by Thomas et al. (2009, 2012) and Van Zomeren et al. (2008; Figure 2).

Like DPMCA, social identity or we thinking system is one of the key variables in team building and collective actions to address different challenges such as ASR. However, in this model social identity is more important and it is believed that it can influence the effects of participative efficiency and negative emotions on collective behaviors (Bamberg et al., 2015). Negative emotions refer to very strong reactions of people (e.g., anger, sadness, etc.), and situations such as injustice and inequality (Kessler and Hollbach, 2005; Thomas et al., 2009). Collective efficiency also refers to the feeling of individuals towards usefulness and efficiency of collective and group actions in solving a problem or crisis (Van Zomeren et al., 2010). In addition to indirect effects, the variables of collective efficiency and negative emotions, through the mediating role of social identity, have indirect effects on intentions towards collective action (Thomas and McGarty, 2009; Bamberg et al., 2015; Figure 2).

## Research methodology

### Study area

The present study was conducted in Fashafuyeh plain. With an area of about 330 square kilometers, this plain, being located in the south of the capital of Iran (Tehran), is positioned on the bed of the Karaj River. The average annual precipitation and evapotranspiration are 155 and 1,061 mm, respectively. This plain covers the city of Robat Karim, Imam Khomeini International Airport and 20 more villages. Geologically, the maximum aquifer thickness exceeds 150 meters.

### Population and sampling

The statistical population consisted of farmers in Fashafuyeh plain in Tehran province (N = 2,400). According to Krejcie and Morgan (1970) sampling table, 331 farmers were selected as the minimum sample. The sample were selected using a five-step and stratified sampling method. In the first stage of sampling, the study area, based on the National Division Guide, was divided into 20 villages. In the second stage, the names and characteristics of the villages of Fashafuyeh plain were determined. In the third stage, the number of farmers in each village was extracted from the statistical handouts of the agricultural Jihad Administration of Robat Karim. In the fourth stage, the total sample size was proportionally distributed among the studied villages. Finally, the sample was randomly selected according to farmer population of each of the villages.

### Research instrument

The research instrument used was a closed-ended questionnaire that used the opinions of a group of academic experts in the technical and social fields of water resource management and also the opinions of practitioners of sustainable water resource management interventions; thus, the face validity and content validities of which were confirmed. After some modifications, the questionnaire was evaluated using a pilot test. The reliability of the tool was examined using criteria including Cronbach’s alpha coefficients, items’ composite reliability, corrected item-total correlation coefficients, and loading factors of measurement models for the variables. Cronbach’s alpha coefficients were calculated after a pilot study and interviews with 30 farmers. The pilot study resulted in some insightful lessons; besides, it implied that all alpha coefficients were greater than the acceptable value of 0.7. The main survey was performed following the pilot study; after the survey, the composite reliability (CR) and the corrected item-total correlation (CITC) coefficients were examined. The values obtained for CR and CITC coefficients were higher than the valid values of 0.7 and 0.3, respectively. Calculation of the loading factors for the measurement models of each composite constructs demonstrated that all their values were greater than the logical value of 0.4.

To appraise the validity of the cross-sectional survey’s questionnaire, convergent or average variance (CV), or Average Variance Extracted (AVE) and divergent validity indices were employed. To evaluate the divergent validity of the research tool in SEM, we first used the average shared squared variance (ASV) index; then, the maximum shared squared variance (MSV) was applied as a supporting criterion.

### Study variables

Generally, there were seven variables in the two frameworks of collective action that were applied to analyze farmers’ participation in ASR management. The method of measuring and operationalizing these variables has been thoroughly presented in Table 1; nevertheless, their conceptual definitions are presented below. All of these variables were measured using a five-level Likert scale (strongly agree to strongly disagree).

**Table 1:** Survey items and variables.

Var.	No	Items	Source
CEPASRM	Collective efficacy of participation in ASR management (CEPASRM)		
	1	Collective action can play a significant role in storing and rehabilitating groundwater resources and reducing water quantity and quality problems.	Self-developed
	2	Group-based activities increase the efficiency of the ASR operations.	
	3	Collective action and community participation are of the main drivers of succeeding ASR plan in the region.	
SNPASRM	Subjective norms of participation in ASR management (SNPASRM)		
	1	People around me and my acquaintances think that I should be involved in ASR operations.	Self-developed
	2	My commitment to participate in ASR management leads to my endorsement by those around me.	
	3	My friends and acquaintances expect me to participate in ASR management in the Fashafuyeh plain.	
NEPASRM	Negative emotions about participation in ASR management (NEPASRM)		
	1	I do not think other stakeholders (farmers, government, and the private sector) will be involved in ASR management.	Self-developed
	2	I am upset if some of the stakeholders do not participate in the various stages of the project or have no commitment to it.	
	3	Violation of any of the stakeholders in the rules and regulations set for the process of the aquifer storage, causes me to violate the rules.	
IPASRM	Intention towards participation in ASR management (IPASRM)		
	1	I want to participate in ASR management in this region.	Self-developed
	2	I would like to pay for ASR management in the area.	
	3	I intent to encourage other farmers to participate in ASR management in Fashafuyeh plain	
	4	If necessary, I would like to learn the skills needed to participate in ASR management.	
	5	I intent to work with experts and specialists of ASR management.	
SIPASRM	Social identity towards participation in ASR management (SIPASRM)		
	1	I would be happy to participate as a member of a group in ASR management.	Self-developed
	2	Participating and playing a role in ASR management is an important part of my self-image.	
	3	I feel I have strong connections with the people involved in ASR management.	
APASRM	Attitude towards participation in ASR management (APASRM)		
	1	In my opinion, participation in ASR management has favorable results for all residents of the plain.	Self-developed
	2	Participating in ASR management is a public duty or wise task to improve current situation.	
	3	Participation in ASR management has so many economic, social, and environmental benefits.	
	4	Farmers’ participation in ASR management in the current water shortage crisis of the plain is a must.	
	5	ASR management in the Fashafuyeh plain is a suitable solution to deal with the crisis, and we farmers and ranchers must be actively involved in this process.	
PBCPASRM	Perceived behavioral control towards participation in ASR management (PBCPASRM)		
	1	I think it is easy to participate in ASR management.	Self-developed
	2	I have enough time and skills to participate in ASR management.	
	3	I have the economic capacity to participate in ASR management.	
	4	I have the sufficient knowledge to participate in ASR management.	
	5	The people of this region have enough ability and capability to participate in ASR management.	

*Intention towards participation in ASR management (IPASRM)*: Indicates the farmers’ willingness to participate in collective actions of ASR management.

*Social identity towards participation in ASR management (SIPASRM)*: Refers to farmers’ identity-oriented beliefs about their participation in collective actions of ASR management.

*Subjective norms of participation in ASR management (SNPASRM)*: Refers to the farmers’ perceptions of the way those surrounding them think about their participations or non-participations in collective ASR management.

*Perceived behavioral control towards participation in ASR management (PBCPASRM)*: Represent the farmers’ perception of their ability to participate in collective ASR management.

*Attitude towards participation in ASR management (APASRM)*: Stands for the positive or negative orientation of the farmers towards participation in ASR management.

*Collective efficacy of participation in ASR management (CEPASRM)*: Indicates the degree of individual belief in the effectiveness of collective action in ASR management.

*Negative emotions about participation in ASR management (NEPASRM)*: Refers to the strong reactions of farmers (anger, resentment, and etc.) to the lack of participation of other farmers and stakeholders in ASR management.

### Data collection and cross-sectional survey

To gather the needed data, face-to-face interview with farmers in Fashafuyeh plain was conducted. In general, 331 questionnaires were distributed, out of which 330 questionnaires were collected. A single questionnaire, owing to many deficiencies in the respondent’s answers, was discarded. Eventually, 330 questionnaires were analyzed; for this purpose, SPSS_26_ and AMOS_25_ were employed.

### Data analysis techniques

Mardia’s coefficients of skewness and kurtosis were employed to test the normality of the data. The estimates of measurement and structural models were used to analyze the data using SEM.

## Results and discussion

### Correlations among the variables

According to the theoretical frameworks, the results of correlation relationships between the variables showed that in DPMCA, four variables of APASRM (*r* = 0.781; *p* < 0.01), SNPASRM (*r* = 0.418; *p* < 0.01), PBCPASRM (*r* = 0.700; *p* < 0.01), and SIPASRM (*r* = 0.486; *p* < 0.01) have positive and significant correlations with IPASRM (Table 2). These results demonstrate that by increasing or amplifying these four variables, IPASRM increases either. Comparison of the correlation values indicates that despite the significance of them, compared to the other two variables (SIPASRM and SNPASRM), APASRM and PBCPASRM have a remarkably stronger correlation with IPASRM. Researchers such as Bamberg et al. (2015) and Fritsche et al. (2018) have supported these findings with their results. In order to elaborate the comparison of the results of present study with the results of previous researchers, its should be mentioned that Bamberg and his colleagues’ results revealed that attitude towards collective climate actions, perceived behavioral control, subjective norms of collective climate actions, and social identity of collective climate actions have significant positive effects on the cooperatives members’ intention towards participation in collective climate actions. Fritsche et al. (2018) also concluded that variables social identity, perceived behavioral control about the collective actions, and attitude towards collective actions are positively and significantly correlated with the variable intention. Based on the results, CEPASRM (*r* = 0.555; *r* < 0.01) and SIPASRM (*r* = 0.486; *r* < 0.01) in EMSICA had positive and significant correlations with IPASRM. The results of the research done by Van Zomeren et al. (2008) confirm these findings. Van Zomeren et al. (2008) employed EMSICA to conceptualize the intention towards collective actions. Their results revealed that the constructs collective efficacy and social identity are of great and positive effect on intention of the individuals to participate in a specific behavior. In contrast, NEPASRM (*r* = 0.413; *p* < 0.01) significantly correlated with IPASRM. This result reflects this insight that with the increase of NEPASRM, IPASRM decreases and vice versa. Similar results can be found among the findings of Thomas et al. (2012) and Faghani et al. (2022). More specifically, Thomas et al. (2012) in their study concluded that negative emotions have significant and negative effects on the intention of the individuals towards taking a specific action. Furthermore, Faghani and his colleagues studied the pro-environmental behaviors of environmental cooperatives in Iran and concluded that negative emotions towards participation in collective pro-environmental behaviors are significantly and negatively correlated with the intention of the cooperative members towards participation in collective pro-environmental behaviors. In addition, the correlation between CEPASRM (*r* = 0.465; *p* < 0.01) and NEPASRM (*r* = −0.341; *p* < 0.01) with SIPASRM was significant. However, since the direction of correlation between them is opposite, the direction of their effects would be different.

**Table 2:** Correlation matrix of the study variables.

	IPASRM	SIPASRM	SNPASRM	PBCPASRM	APASRM	CEPASRM	NEPASRM
IPASRM	1						
SIPASRM	**0.486** ^**^	1					
SNPASRM	**0.418** ^**^	0.300^**^	1				
PBCPASM	**0.700** ^**^	0.417^**^	0.374^**^	1			
APASRM	**0.781** ^**^	0.485^**^	0.434^**^	0.711^**^	1		
CEPASRM	**0.555** ^**^	**0.465** ^**^	0.406^**^	0.507^**^	0.591^**^	1	
NEPASRM	**−0.413** ^**^	**−0.341** ^**^	−0.377^**^	−0.432^**^	−0.513^**^	−0.469^**^	1

IPASRM, Intention towards participation in ASR management; SIPASRM, Social identity towards participation in ASR management; SNPASRM, Subjective norms of participation in ASR management; PBCPASRM, Perceived behavioral control towards participation in ASR management; APASRM, Attitude towards participation in ASR management; CEPASRM, Collective efficacy of participation in ASR management; NEPASRM, Negative emotions about participation in ASR management.

** Sig. level: 0.01 error. The bold values represent the correlations of the theoretical frameworks' variables.

### The results of measurement models in SEM

The results of examining the measurement models of the variables in the two frameworks indicated that for all items, the numerical values of loading factors were greater than or equal to 0.4; Therefore, loading factors had acceptable values (Table 3). The values obtained for CR and AVE were greater than or equal to 0.7 and 0.5, respectively (Valizadeh et al., 2021b). These results also show that the different parts of the tools developed and applied in the present study fulfilled the composite reliability and convergent validity criteria; besides, Comparison of divergent validity indices (ASV and MSV) with AVE index revealed that the values of these indices were less than AVE values. Therefore, it can be concluded that the study tool had divergent validity. The last two columns in Table 3 stand for the values of Mardia’s coefficients of multivariate skewness and kurtosis. Given that their values were less than ±1.96, it can be concluded that the research data had normal distribution (Table 3).

**Table 3:** Th results of estimating measurement model and reliability and validity assessment.

Items/index	IPASRM	SIPASRM	SNPASRM	PBCPASRM	APASRM	CEPASRM	NEPASRM	Skew	Kurtosis
IPASRM1	0.86*							0.298	0.725
IPASRM2	0.54							1.525	0.856
IPASRM3	0.84							0.396	0.991
IPASRM4	0.50							−0.722	0.687
IPASRM5	0.59							1.235	1.365
SIPASRM1		0.75*						−0.459	0.775
SIPASRM2		0.80						1.991	0.288
SIPASRM3		0.40						0.753	−0.163
SNPASRM1			0.79*					0.168	0.240
SNPASRM2			0.78					−1.750	1.927
SNPASRM3			0.75					0.456	−0.746
PBCPASRM1				0.80*				0.258	0.510
PBCPASRM2				0.76				0.368	0.752
PBCPASRM3				0.45				1.815	−1.249
PBCPASRM4				0.60				−0.753	0.306
PBCPASRM5				0.56				1.685	0.466
APASRM1					0.58*			1.202	0.726
APASRM2					0.66			0.287	−0.377
APASRM3					0.68			−0.695	1.758
APASRM4					0.78			0.367	0.805
APASRM5					0.75			0.741	−0.230
CEPASRM1						0.80*		0.984	−0.462
CEPASRM2						0.78		0.583	1.711
CEPASRM3						0.57		−1.235	0.631
NEPASRM1							0.92*	0.369	−1.025
NEPASRM2							0.70	−0.613	0.522
NEPASRM3							0.89	1.586	0.699
CR	0.89	0.83	0.83	0.90	0.90	0.83	0.79		–
AVE	0.63	0.64	0.62	0.67	0.66	0.63	0.59	–	–
MSV	0.60	0.23	0.18	0.50	0.60	0.35	0.26	–	–
ASV	0.33	0.18	0.14	0.29	0.36	0.25	0.18	–	–

* Fixed item in confirmatory factor analysis.

### Results of structural models and comparison of their estimates

**Figure 3:**
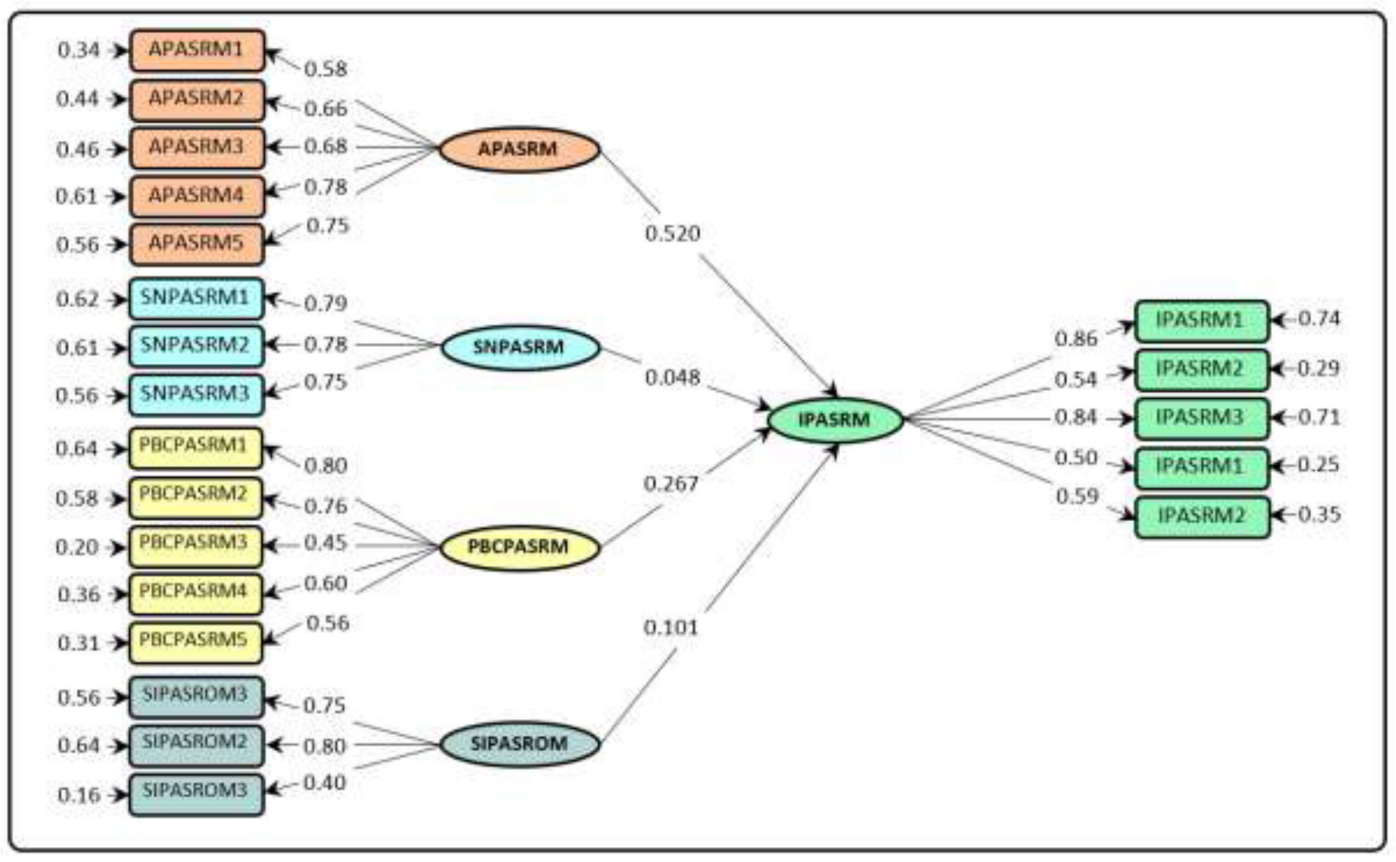
Structural model of DPMCA.

Running and estimation of DPMCA structural model showed that APASRM (*β* = 0.520; *p* < 0.01), PBCPASRM (*β* = 0.267; *p* < 0.01), and SIPASRM (*β* = 0.101; *p* < 0.01) had positive and significant effects on IPASRM (Table 4; Figure 3). This result indicates the high power of these variables in explaining and directing IPASRM. Of these three variables, APASRM and PBCPASRM had stronger standardized effects than SIPASRM. Nevertheless, the role of SIPASRM in explaining IPASRM cannot be ignored. It should be mentioned that in estimating the DPMCA structural model, the effect of SNPASRM on IPASRM was not significant. Therefore, the SNPASRM→IPASRM path or hypothesis was not supported (*β* = −0.048; n.s.). Taken together, the independent variables were able to predict 65.4% of the variance changes of IPASRM in DPMCA (Table 4; Figure 3).

**Table 4:** The results of structural models and comparison of the results of two models.

Model	Hypothesized relationship	Unstandardized estimates	S.E.	Standardized estimates	Sig.	Hypothesis test
DPMCA	APASRM → IPASRM	0.507	0.050	0.520	0.001	Supported
	SNPASRM → IPASRM	0.081	0.063	0.048	0.200	Unsupported
	PBCPASRM → IPASRM	0.301	0.054	0.267	0.001	Supported
	SIPASRM → IPASRM	0.185	0.071	0.101	0.009	Supported
EMSICA	CEPASRM → IPASRM	0.527	0.091	0.336	0.001	Supported
	SIPASRM → IPASRM	0.432	0.100	0.235	0.001	Supported
	NEPASRM → IPASRM	−0.236	0.068	−0.176	0.001	Supported
	CEPASRM → SIPASRM	0.725	0.080	0.463	0.001	Supported
	NEPASRM → SIPASRM	−0.269	0.069	−0.200	0.001	Supported

IPASRM, Intention towards participation in ASR management; SIPASRM, Social identity towards participation in ASR management; SNPASRM, Subjective norms of participation in ASR management; PBCPASRM, Perceived behavioral control towards participation in ASR management; APASRM, Attitude towards participation in ASR management; CEPASRM, Collective efficacy of participation in ASR management; NEPASRM, Negative emotions about participation in ASR management.

**Figure 4:**
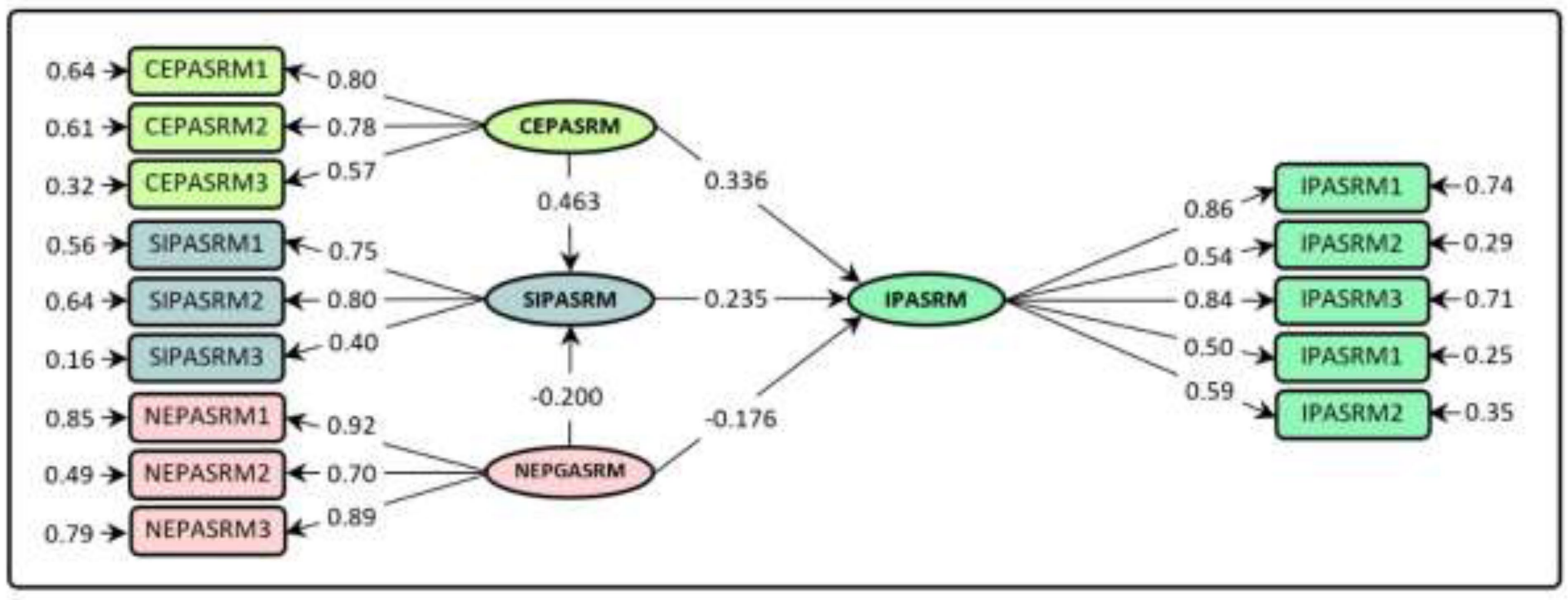
Structural model of EMSICA.

Estimation of EMSICA structural model revealed that all predicted paths were significant. In other words, all the hypotheses predicted in EMSICA were confirmed (Table 4; Figure 4). Based on the results, the standardized effects of CEPASRM (*β* = 0.336; *p* < 0.01) and SIPASRM (*β* = 0.235; *p* < 0.01) on IPASRM were positive and significant; that is, with increasing and reinforcing these variables, IPASRM is strengthened. However, NEPASRM (*β* = −0.176; *p* < 0.01) affected IPASRM negatively. This variable had a significant negative effect on SIPASRM (*β* = −0.200; *p* < 0.01). CEPASRM (*β* = 0.463; *p* < 0.01) was the second variable that was predicted to have a positive and significant effect on SIPASRM in EMSICA. And the results of structural model estimates confirmed this hypothesis. In general, the results indicated that the independent variables in EMSICA could account for about 36.5 and 33.5% of the changes in IPASRM and SIPASRM, respectively (Table 4; Figure 4).

### Estimation of indirect effects and total independent variables on the dependent variable in EMSICA

Due to the lack of a mediating variable in the DPMCA, a direct/total structural model was employed to run that; therefore, there were no indirect effects for the independent variables. Though, in EMSICA, the SIPASRM acts as a mediating variable, and therefore a mediation/indirect structural model was applied in its running process. The results of employing the mediation/indirect structural model helped to estimate the indirect and total (causal) effects of independent variables on IPASRM (Table 5). The results obtained from this section showed that CEPASRM has the greatest indirect effects; however, the indirect effect of NEPASRM on IPASRM is not significant. In addition, the variables CEPASRM, SIPASRM, and NEPASRM had the largest total/causal effects on IPASRM, respectively.

**Table 5:** The results of indirect and total effects on IPASRM in the EMSICA.

Model	Variables	Direct estimate (DE)	Indirect estimates	Total effects
EMSICA	CEPASRM	0.336	0.108	0.444
	SIPASRM	0.235	----	0.235
	NEPASRM	−0.176	−0.047	−0.223

IPASRM, Intention towards participation in ASR management; SIPASRM, Social identity towards participation in ASR management; CEPASRM, Collective efficacy of participation in ASR management; NEPASRM, Negative emotions about participation in ASR management.

### Results from estimation of fitness and validation indices for the structural models

In order to evaluate the fitness of structural models, seven evaluation criteria including Comparative fit index (CFI), Goodness of fit index (GFI), Adjusted Goodness of Fit Index (AGFI), Normed fit index (NFI), Incremental Fit Index (IFI), Root Mean Square Error of Approximation (RMSEA), and chi-square normalized by degrees of freedom (CMIN/DF) were applied. Acceptable obtained values for the two structural models tested in this study have been presented in Table 6. The Comparison of the fitness criteria obtained for DPMCA with acceptable values shows that the model is of suitable validity; Besides, the fitness criteria confirmed the validity of EMSICA (Table 6).

**Table 6:** Results from estimation of fitness indices for the structural models.

Fitness index	Cut-offs	Results for present study	
		DPMCA	EMSICA
CFI	≥0.90	0.938	0.913
GFI	≥0.90	0.922	0.906
AGFI	≥0.90	0.935	0.919
NFI	≥0.90	0.940	0.925
IFI	≥0.90	0.926	0.937
RMSEA	≤0.10	0.063	0.058
chi-square normalized by degrees of freedom	≤5	2.863	3.759

## Conclusion and policy implications

The main purpose of the present study was to analyze the intentions towards participation in ASR management, using two EMSICA and DPMCA frameworks. The results demonstrated that in DPMCA, APASRM is the strongest determinant of IPASRM. According to DPMCA, farmers’ positive attitudes toward participation in collective ASR management measures, highly depend on the understanding of the positive consequences of their participation. In other words, farmers should recognize that by participating and taking collective actions for ASR management, they (as the agricultural community) and the whole region would have tangible benefits in terms of water, agriculture, and economy. Therefore, it is recommended that some local training workshops and courses get arranged for farmers in this area. It is worth mentioning that in these training course,s the outcomes and benefits of farmers’ participation in collective measures of ASR management shall be addressed and emphasized. The main outcomes and benefits are including solving agricultural water shortage problems, preventing migration, raising groundwater level, reducing water pollution, and producing healthy agriculture crops. In fact, it should be kept in mind that each of the aforementioned points should be supported with valid reference to the scientific contents. (which have become understandable messages for farmers). The results of running DPMCA also revealed that PBCPASRM positively and significantly affected IPASRM. This result indicated that according to the DPMCA social identity model, farmers believed that participation in the collective actions related to ASR management was not beyond their capability and control; that is, they have reached a desirable level of self-confidence in their ability to participate in the collective actions of ASR management, and this self-confidence has a positive effect on their IPASRM. Such a result might be recognized as a turning point for sustainable water resources management programs, interventionists and managers/policy-makers of ASR management. Because, in such circumstances, there is no need to spend much time and financial or human resources for strengthening the self-efficacy and self-confidence of farmers, so that they can participate in collective actions of ASR. The insignificance of the effect of SNPASRM on IPASRM supports such a conclusion either. This result shows that farmers in their decisions to participate in collective ASR management activities, are not influenced by possible beliefs dictated by others.

CEPASRM was the strongest direct determinant of IPASRM in EMSICA, which affected that positively. From this result, it can be inferred that farmers’ beliefs about the effectiveness of their collective and participatory actions might play an important role in increasing their intentions and motivations to participate in ASR management. In other words, if farmers conclude that collective actions of ASR management can have significant and positive consequences for improving water-related crises in the region, then they would be more willing to participate in that action. They may even spontaneously initiate such collective actions and encourage other organizations and stakeholders to play more active roles. One of the best strategies to raise awareness and enlightenment on the effectiveness of collective actions in ASR management is the application of successful national and international experiences of collective/social environmental crisis management actions. In this regard, it is suggested that the institutions responsible for the implementation of sustainable water management programs use such strategies to form this belief that relying on *collective will* and the effectiveness of participatory measures in projects such as ASR management can overcome many problems.

NEPASRM negatively and significantly affected IPASRM. It can be concluded that beliefs, feelings, and reactions such as anger, pessimism, etc. towards collective actions in the field of ASR management can reduce farmers’ willingness towards participation. Considering that the formation of such negative feelings about participation is generally related to farmers’ previous and personal experience of disputes with various stakeholders (especially governmental stakeholders), lack of mutual trust, and stakeholders’ effort to maximize their share of the collective interest, it is recommended that the share of participation, decision-making power, authority, job descriptions, etc. be specified accurately in the implementation of ASR management. Furthermore, it is recommended that each stakeholder involved in the collective ASR management, provide an executive guarantee/commitment to perform his/her duties. This would build trust in the agricultural community. Building trust also reduces negative emotions and strengthens IPASRM. Based on the results of running DPMCA and EMSICA frameworks, SIPASRM affected IPASRM positively and significantly. This suggests that both social identity models of collective action see SIPASRM as an undeniable requirement for farmers’ willingness to participate in collective ASR management. In other words, in order to strengthening IPASRM, they need to find an identity and feel that the group members and their *personal selves* have no meaning without each other. In such a situation, the individual interests of each farmer would be in line with the collective interests of the group involved in ASR management. Such convergence in the individual and group interest of farmers might have a synergetic and constructive effect on IPASRM and effectiveness of ASR management. In this regard, it is recommended that policy-makers, planners, managers, and practitioners of sustainable water resources management programs (like ASR management projects) use the participation and consensus of farmers in various stages of the projects such as diagnosis and contextualization of the problems, plannings, implementations, and evaluations. This would lead to the formation of a social identity and then they would consider ASR management project as their own; therefore, achieving collective goals would be equivalent to achieving individual goals. Such a collective and *we thinking system* would ultimately strengthen IPASRM and the effectiveness of policies and programs.

The main take-home massage of the present research is that for the first time in the world, social identity models of collective actions have been used in conceptualizing IPASRM. The results of this study showed that social identity models of collective action have a good capability in predicting IPASRM; though, DPMCA has more explanatory power than EMSICA. This study introduces the formation of a *collective and we thinking system,* as a significant basis of any policy, program, and intervention in the field of sustainable ASR and water resource management, in agricultural communities.

This research had three limitations that should be mentioned and suggestions for future researchers should be made based on them. First, in this study, the self-reporting system was used to collect the required data, and the results were interpreted based on the self-reports of the respondents in Fashafuyeh plain. Future researchers can use non-self-reporting methods such as case studies, focus groups, etc. to collect similar information. This can help increase the plausibility of research results. Second, in this study, in order to strengthen the feasibility of the research process, simplified versions of social identity models of collective action were used. Future researchers can develop these models by adding other social and cultural variables. Thirdly, this study was conducted only in Fashafuyeh plain and its results can be used in other geographical areas that have common characteristics with this plain. However, in other geographical areas that have different characteristics, separate researches should be conducted. Conducting such researches can help to cross-validate the results of the current research and increase their generalizability. In this regard, it is suggested that future researchers use social identity models of collective action to manage groundwater water resources in other plains.

## Data availability statement

The original contributions presented in the study are included in the article/Supplementary material, further inquiries can be directed to the corresponding author.

## Ethics statement

Ethical review and approval was not required for the study on human participants in accordance with the local legislation and institutional requirements. Written informed consent from the patients/participants or patients/participants legal guardian/next of kin was not required to participate in this study in accordance with the national legislation and the institutional requirements.

## Author contributions

All authors listed have made a substantial, direct, and intellectual contribution to the work and approved it for publication.

## Conflict of interest

The authors declare that the research was conducted in the absence of any commercial or financial relationships that could be construed as a potential conflict of interest.

## Publisher’s note

All claims expressed in this article are solely those of the authors and do not necessarily represent those of their affiliated organizations, or those of the publisher, the editors and the reviewers. Any product that may be evaluated in this article, or claim that may be made by its manufacturer, is not guaranteed or endorsed by the publisher.
